# Clonal amplification and maternal-infant transmission of nevirapine-resistant HIV-1 variants in breast milk following single-dose nevirapine prophylaxis

**DOI:** 10.1186/1742-4690-10-88

**Published:** 2013-08-14

**Authors:** Sallie R Permar, Maria G Salazar, Feng Gao, Fangping Cai, Gerald H Learn, Linda Kalilani, Beatrice H Hahn, George M Shaw, Jesus F Salazar-Gonzalez

**Affiliations:** 1 Duke Human Vaccine Institute, Duke University Medical Center, Durham, NC, USA; 2 Department of Medicine, University of Alabama at Birmingham, Birmingham, AL 352941, USA; 3 Department of Medicine, Perelman School of Medicine, University of Pennsylvania, Philadelphia, PA, USA; 4 College of Medicine, University of Malawi, Blantyre, Malawi

**Keywords:** Mother-to-child transmission, Breast milk, HIV-transmission, Nevirapine, Drug-resistant variant, K103N, Transmitted virus, Clonal amplification, Antiretroviral prophylaxis

## Abstract

**Background:**

Intrapartum administration of single-dose nevirapine (sdNVP) reduces perinatal HIV-1 transmission in resource-limiting settings by half. Yet this strategy has limited effect on subsequent breast milk transmission, making the case for new treatment approaches to extend maternal/infant antiretroviral prophylaxis through the period of lactation. Maternal and transmitted infant HIV-1 variants frequently develop NVP resistance mutations following sdNVP, complicating subsequent treatment/prophylaxis regimens. However, it is not clear whether NVP-resistant viruses are transmitted via breastfeeding or arise *de novo* in the infant.

**Findings:**

We performed a detailed HIV genetic analysis using single genome sequencing to identify the origin of drug-resistant variants in an sdNVP-treated postnatally-transmitting mother-infant pair. Phylogenetic analysis of HIV sequences from the child revealed low-diversity variants indicating infection by a subtype C single transmitted/founder virus that shared full-length sequence identity with a clonally-amplified maternal breast milk virus variant harboring the K103N NVP resistance mutation.

**Conclusion:**

In this mother/child pair, clonal amplification of maternal NVP-resistant HIV variants present in systemic and mammary gland compartments following intrapartum sdNVP represents one source of transmitted NVP-resistant variants that is responsible for the acquisition of drug resistant virus by the breastfeeding infant. This finding emphasizes the need for combination antiretroviral prophylaxis to prevent mother-to-child HIV transmission.

## Introduction

In 2011, 330,000 infants acquired HIV infection from their mothers [1]. Most of mother-to-child transmissions (MTCT) occur in low- and middle-income countries, where HIV transmission through breastfeeding accounts for 30–50% of infant infections in the absence of antiretroviral (ARV) prophylaxis [2]. In sub-Saharan Africa, formula feeding is not a recommended alternative due to its association with increased morbidity and mortality, caused by malnutrition, respiratory diseases and diarrheal complications [3]. Furthermore, several studies have substantiated the benefit of breastfeeding over formula feeding, despite the risk of breast milk transmission [4-6]. WHO currently recommends exclusive breastfeeding for the first 6 months of life, followed by complementary foods and breastfeeding until 12 months of age, accompanied by postnatal infant or maternal antiretroviral prophylaxis to reduce HIV transmission during breastfeeding [1]. Initial studies showed that intrapartum single-dose nevirapine (sdNVP) prophylaxis reduced the risk of MTCT by half [7] but lacked efficacy to prevent breast milk HIV transmission. More recently, extended NVP prophylaxis regimens reduced breast milk transmission when compared to sdNVP [8]. Though convenient, inexpensive, and effective, sdNVP prophylaxis selects for NVP-resistant (NVP-R) variants in a high proportion of women (19–75%) and their infected infants (33–87%) [9,10] and these variants remain detectable for a year or more [10-12]. Moreover, NVP-R variants emerge more frequently and persist longer after exposure to extended NVP prophylaxis [13,14], while sdNVP administration increases the risk of virologic failure to subsequent ARV treatment [15].

Although a large fraction of infants harbor NVP-R variants following perinatal NVP administration, it is not clear whether NVP-R variants are transmitted to the infant postnatally or if they arise *de novo* in the infant [9-11,16]. Previous studies have not rigorously examined the drug-resistant profile of transmitted/founder (T/F) virus (es) that are responsible for transmission and productive clinical infection in the infant [10,11,13,14,16]. Understanding the precise origin of drug-resistant strains in MTCT may aide in the design of improved, more broadly effective prophylaxis regimens that will not impair future treatment options for infected infants. Our previous study of chronically HIV-infected Malawian lactating women who received intrapartum sdNVP revealed continual trafficking of blood-derived viral variants into the mammary glands followed by transient local replication of some variants that disproportionally contributed to low-diversity (clonally-amplified) viral populations in breast milk [17]. Here we hypothesized that clonally-amplified variants in breast milk are selected for drug-resistance by NVP and that they are likely to be transmitted to the infant due to their proportional abundance and selective replication fitness in the presence of NVP. To test this hypothesis, we conducted a longitudinal genetic and drug-resistant mutation analysis of HIV variants in plasma and milk of a postnatal-transmitting mother-infant pair following sdNVP administration.

## Findings

### Postnatal transmission of a single virus and time estimates of infection by HIV sequence diversity analysis in the infant

We studied a chronically subtype C HIV-infected lactating Malawian woman (subject 4403) who received intrapartum sdNVP prophylaxis (200 mg) to prevent MTCT the day before delivery, gave birth to an uninfected child and was followed longitudinally for 12 weeks (Table 1, Additional file 1). The child, subject 4419, received a NVP dose at birth (2 mg NVP/Kg of weight), tested blood HIV DNA PCR negative at birth and four weeks of age and continued to be breastfed by the mother until 6 months of age. At 12 weeks of age, the child had a positive blood HIV DNA PCR, and high plasma viral load (1,210,000 copies/ml) strongly supporting breast milk transmission with the two previous negative tests. Under these circumstances, a potentially transmitted NVP-R virus may have a fitness advantage over wild-type virus with NVP-R variant being able to establish productive infection in the setting of the long life of NVP. The infant subsequently died at 6 months of age of respiratory complications, despite trimethoprim-sulfamethoxazole prophylaxis. Phylogenetic analysis of multiple envelope (*env*) sequences derived from child’s plasma at the time of HIV diagnosis (Additional file 1) revealed low-diversity sequences (mean nucleotide diversity: 0.10%, range: 0.00–0.20%) suggesting recent infection by a single virus. Indeed, *env* sequences conformed to predictions of a mathematical model of random evolution by a single virus [18]; as sequences exhibited a Poisson distribution of mutations and star-like phylogeny, which coalesced to an inferred consensus sequence that identifies the virus present at or near the estimated time of transmission (Table 2). Using Poisson model parameters, the mean *env* divergence time of the 12-week sample (83 days postpartum) since a most recent common ancestor (MRCA) was 43 days (95% confidence intervals [CI]: 32, 54 days), a time consistent with postpartum transmission of a single virus. In addition, when sets of 5’-half and 3’-half infant HIV genomes were analyzed, each set conformed to model predictions, as described above for *env*-only sequences (Table 2). Moreover, time interval estimates since the MRCA overlapped in all three data sets and fell within the time frame of the last HIV PCR negative (4 weeks of age) and first PCR positive test (12 weeks of age) of the infant. Given the relative immaturity of the infant’s immune system during the first few months of life, the low sequence diversity and Poisson distribution of mutations in the viral quasispecies support negligible effect of immune selection pressure of placentally-acquired maternal antibodies. Under conditions of low-selection pressure, time estimates since a MRCA for the infected child using the Poisson model are reliable and comparable to those estimated by Bayesian methods [18]. Accordingly, a Bayesian relaxed clock-based analysis of *env* sequences (Additional file 1) estimated a mean of 54 days (95% highest probability density intervals: 17, 105) since a MRCA, which is slightly higher than the Poisson estimate but consistent with predictions based on the infant HIV-1 testing. Together, these data suggest that the child could have been infected as early as 4.3 weeks or as late as 7.7 weeks of age.

**Table 1:** Clinical and virological data of chronically HIV-infected lactating woman 4403

**Sample ID weeks postpartum**	**Sample date**	**Plasma virus load (copies/ml)**	**Breast milk virus load, left and right (copies/ml)**	**CD4 count (cells/μl)**	ART* prophylaxis	**Infant blood HIV DNA PCR status**
Third trimester of pregnancy	04/25/08	519,000		208		
Parturition	05/24/08	56,200			Maternal sdNVP on 05/23/08	PCR negative
Week 4	06/27/08	100,892	Left: 101,500			PCR negative
			Right: 30,450			
Week 12	08/15/08	2,120	Left: <240			PCR positive

* ART denotes antiretroviral therapy.

**Table 2:** Statistics and mathematical model timing estimates of the most recent common ancestor (MRCA) of the infant’s HIV sequences detected at the time of postnatal HIV diagnosis

**HIV sequence**	**Total number of sequences**	**Maximum length of sequence**	**Maximum HD**	**Mean HD (%)**	**Poisson estimated days since MRCA (95% CI)**	**Lambda**	**Standard deviation**	**Goodness of fit p-value**	**HD fit to poisson**	**Star phylogeny**
*Env*-only	22	2571	7	2.6	43 (32, 54)	2.628	0.334	0.987	Yes	Yes
3’-half	22	4682	10	4.3	38 (31, 46)	4.264	0.426	0.960	Yes	Yes
5’-half	21	5349	9	4.7	37 (30, 44)	4.657	0.447	0.225	Yes	Yes

### The inferred infant T/F HIV genome is closely related to a population of low-diversity variants in maternal breast milk near the predicted time of infection

**Figure 1:**
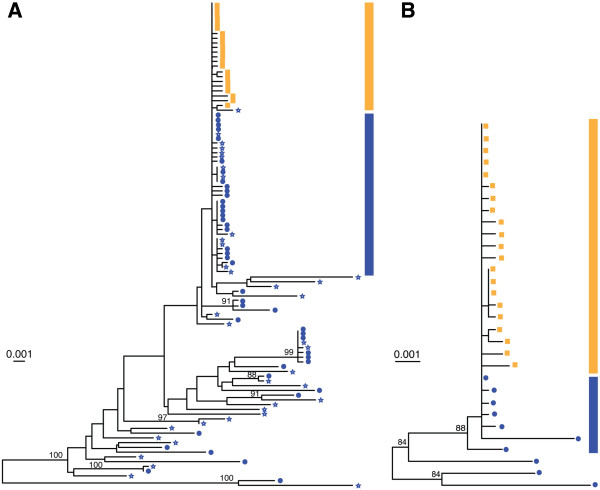
**Maximum-likelihood trees of *env *and *pol *genes from 4403–4419 MTCT pair.** Maternal breast milk virus *env***(A)** and *pol***(B)** sequences (blue stars = right breast; blue circles = left breast) were obtained from a sample collected at 4 weeks postpartum and infant virus *env***(A)** and *pol***(B)** sequences (orange squares) were derived from a plasma sample collected at 12 weeks of age. Groups of identical/near-identical sequences (defined as sequences with four or fewer nucleotide substitutions compared to consensus sequence) in maternal breast milk (vertical blue bar) or infant plasma (vertical orange bar) are indicated. Greater than 90% of the indicated near-identical sequences had fewer than four substitutions compared to consensus. Numerals at nodes indicate approximate bootstrap support values ≥ 70%. The scale bar represents 0.001 (0.1%) nucleotide substitutions per site.

**Figure 2:**
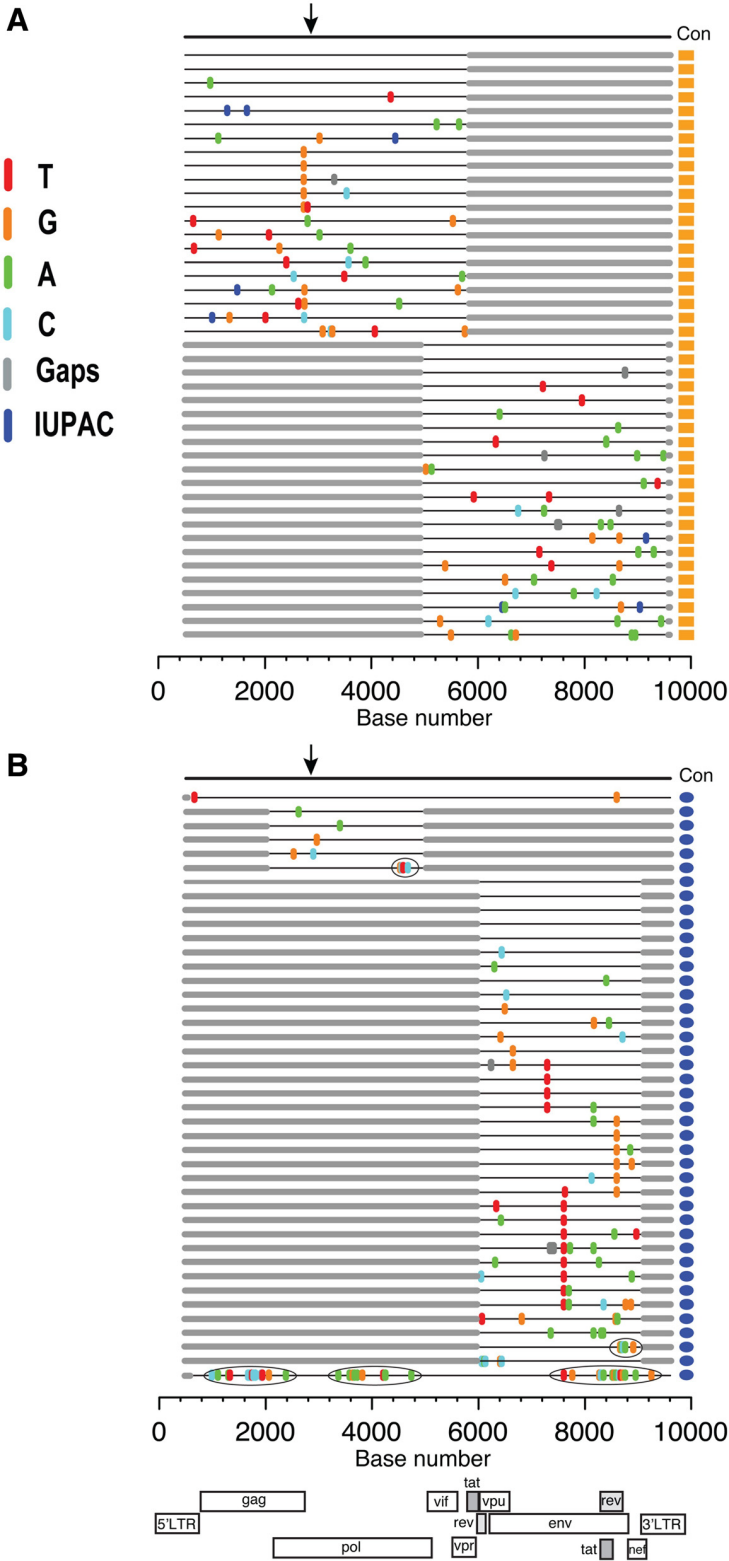
**Highlighter analyses of HIV sequences from infant plasma and maternal breast milk. (A)** Multiple 5’-half and 3’-half genome sequences derived from infant plasma at the time of HIV diagnosis are aligned below the infant consensus sequence (Con). Nucleotide differences from the inferred T/F consensus sequence are indicated by tic marks color-coded for each base. IUPAC denotes International Union of Pure and Applied Chemistry ambiguous base assignments caused by *Taq* polymerase-induced nucleotide misincorporation occurring in the first two cycles of the PCR amplification. Flanking gray boxes indicate regions not amplified. Gray tics indicate deletions. The arrow indicates the location of the K103N mutation in the RT gene in consensus and all SGA sequences (AAA to AAC mutation). The horizontal axis indicates nucleotide positions based on HXB2 reference sequence numbering. Consensus sequence begins at nucleotide position 484 in the 5’ long terminal repeat (LTR) U5 and extend to position 9,606 in the 3’ LTR R. **(B)** Two 9 Kb amplicons, and multiple *pol* and *rev*/*env* fragments representing low-diversity breast milk sequences collected at four weeks postpartum (shown at the left of the blue bars in Figure 1) are aligned below the consensus sequence (identical to Con from Figure 1A). The arrow represents the location of the K103N mutation. Clustered mutations enclosed in elipses reflect recombination with more divergent maternal sequences. The horizontal axis indicates HXB2 nucleotide positions. Sequences begin at nucleotide position 582 in the 5’ LTR U5 and extend to position 9,606 in the 3’ LTR R. Boxes corresponding to LTRs and 9 major protein coding regions of the HIV genome are shown at the bottom.

A maximum-likelihood tree clearly showed that the child’s *env* sequences (indicated with a vertical orange bar in Figure 1A) cluster together with a group of low-diversity maternal sequences (vertical blue bar in Figure 1A) present among a genetically heterogeneous virus population derived from breast (left and right) milk collected at 4 weeks postpartum. A similar pattern was observed when we analyzed viral *pol* sequences from mother and child (Figure 1B). The clustering of child and maternal sequences in a single branch of identity and near-identity (using two different HIV genes) revealed the anatomical source of the transmitted virus, and its exact genetic identity. Next, we inferred the full-length nucleotide sequence of the virus that established infection in the child. A highlighter plot of partially overlapping 5’- and 3’-half genomes derived from plasma at diagnosis (12 week of age) show few random mutations with sequences coalescing to a single consensus that represents the T/F virus (Figure 2A). Two sequences in each half genome were identical to the infant consensus virus, while most other sequences were 1, 2, or 3 nucleotides away from the consensus. A subset of viruses exhibited shared polymorphisms not found in maternal sequences that are interpreted as stochastic mutations occurring in the early replication cycles of the founder virus, but with enough fitness to persist as minority variants. Figure 2B show maternal low-diversity HIV (*pol* and *rev*/*env*) sequences from 4 weeks postpartum breast milk (right and left breast for *env* sequences) coalescing to a consensus identical to the infant consensus virus, thus suggesting common ancestry. Most milk sequences differed from consensus by 0, 1, 2, and 3 nucleotides, while sequences with >4 nucleotide changes represented viral recombinants with more divergent virus sequences (mutations clustered within a restricted region of the genome and were present in other phylogenetically distant milk sequences). Clonally-amplified *pol* and *rev*/*env* milk sequences were linked by an amplified 9 Kb milk variant (Figure 2B, sequence below the consensus) that differed from the infant full-length T/F virus by only two nucleotides, indicating common ancestry. Recombination of clonally-amplified milk variants with more divergent viral sequences was substantial; examples of recombination spots are indicated within a circle for three milk sequences including a second 9 Kb amplicon (Figure 2B, bottom sequence). The infant virus and the corresponding clonally-amplified maternal breast milk variant had intact open reading frames for all nine major protein-coding HIV genes, as expected and shown previously for all T/F virus genomes [19]. Together, these data confirm that the mammary gland is the anatomical source of the postnatally-transmitted virus.

### The transmitted virus is a NVP-R variant that underwent prior selection and clonal amplification in the lactating mother

**Figure 3:**
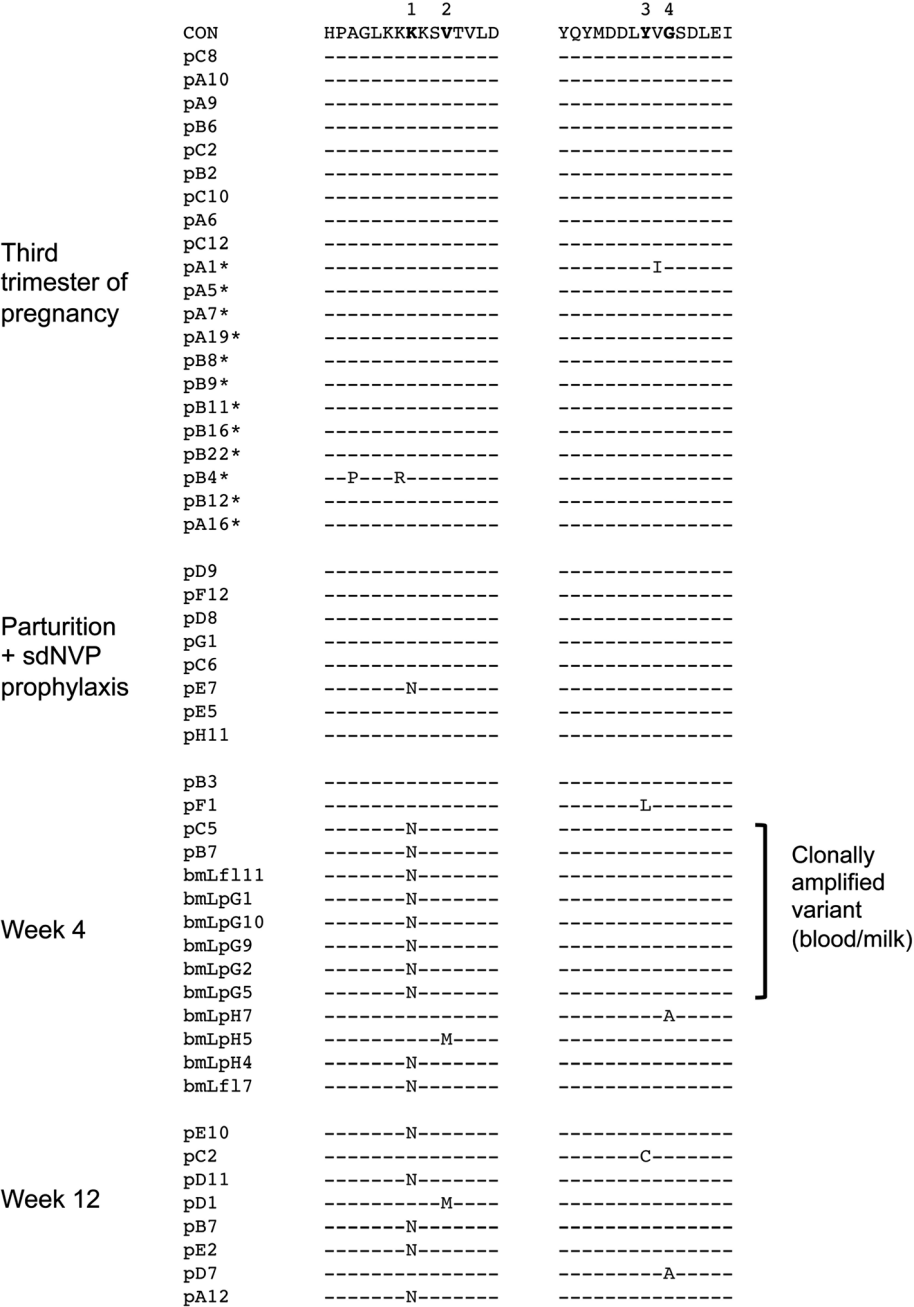
**Selection of HIV-1 RT drug-resistance mutations following ARV drug exposure in maternal plasma samples.** Amino acid alignment of *pol* gene regions indicating single point mutations associated with high-level resistance to ARV drugs in subject 4403. CON denotes amino acid consensus sequence from the earliest maternal plasma sample. Bold amino acid residues in the consensus correspond to selected drug-resistant point mutations. Point mutations numbered 1–4 are as follows: 1 for K103N, 2 for V106M, 3 for Y188C/L, 4 for G190A. Dashes denote identity with consensus, while letters correspond to amino acid changes. Asterisks indicate sequences derived from near end-point diluted PCR positive wells (50% positive PCR wells). All other HIV *pol* sequences were derived by SGA as described [19]. bmL denotes milk sequences derived from left breast; all other sequences are derived from blood plasma. Low-diversity clonally-amplified sequences (0–2 nucleotides away from consensus; bmL_pG5 is a recombinant virus harboring a cluster of 6 nucleotide mismatches shared with more divergent virus sequences) are indicated with brackets.

**Figure 4:**
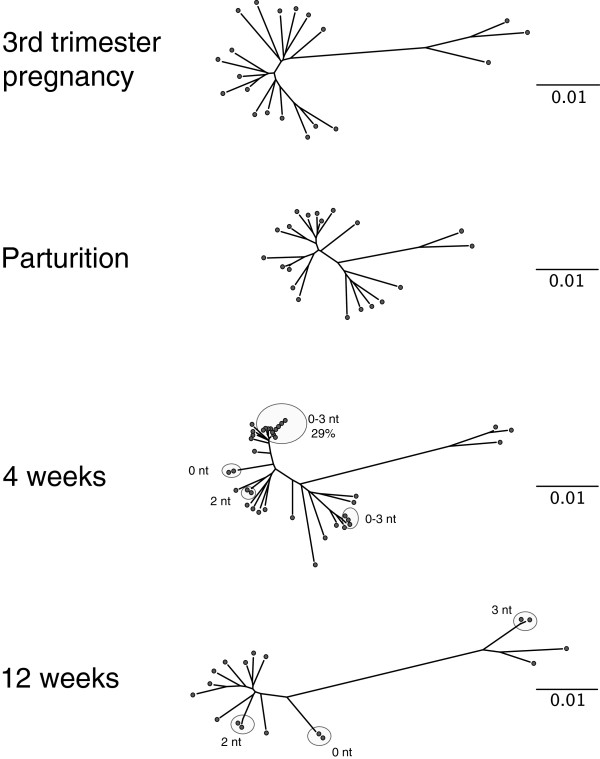
**Neighbor-joining trees of maternal 4403 *env *sequences from longitudinal plasmas samples.** Full-length maternal *env* sequences generated by SGA from third trimester pregnancy and follow-up visits up to 12 weeks postpartum were aligned and plotted as radial Neighbor-joining trees. Clusters of monotypic and low-diversity sequences are encircled; the number of nucleotides by which these sequences differ from MRCA (branch node) is indicated for each cluster. The scale bar represents 0.01 nucleotide substitutions per site (1% divergence).

Because sdNVP prophylaxis is known to select for NVP-R variants [9-11], we investigated drug resistance mutations in the milk and infant virus sequences. We observed that the K103N mutation which confers high-level resistance to NVP [20] was present in all of the infant’s *pol* gene sequences, and hence the T/F virus (marked with an arrow in Figure 2A), as well as in 50% of maternal plasma and 80% of breast milk *pol* sequences (Figure 3). Without exception, *pol* sequences from the major clonally-amplified virus population in both blood (2 out of 2) and mammary (6 out of 6) compartments had the K103N mutation (Figures 2B and 3). Identifying the mechanisms underlying HIV clonal amplifications in breast milk may have important implications in transmission risk and guiding the development of new prophylactic strategies. Major clonally-amplified *env* variants accounted for 35% and 50% of the total breast milk virus population at four weeks postpartum from right (13/37 sequences) and left breast (22/44 sequences), respectively (Figure 1A). Clonally-amplified variants were also present in maternal plasma at 4 weeks postpartum (Figure 4), albeit with slightly lower frequency to that seen in breast milk (29%; 11 out of 38 *env* sequences) as previously shown [17]. Although the K103N mutation was not present in any of 9 SGA and 12 end-point PCR-amplified plasma sequences at 3^rd^ trimester of gestation, one out of eight *pol* sequences derived from maternal plasma collected after delivery (approximately one day after sdNVP) had the K103N mutation (clone pE7). The one-log reduction in plasma viral load that occurred between the 3^rd^ trimester and parturition samples (519,000 vs. 56,200 copies/ml, respectively) was an indication of the antiretroviral response between NPV administration at the onset of labor and sample collection one day later. Although the depth of sequencing was not sufficient to determine whether clone pE7 which was present at parturition preexisted as a low-frequency variant before NVP prophylaxis, mathematical models [21] and a pooled analysis of several clinical studies [22] suggest that virologic failure is most likely caused by the preexistence of low-frequency resistant mutants. Four weeks after delivery, wild-type NVP-R susceptible virus was nearly replaced with variants carrying individual non-nucleoside reverse transcriptase inhibitor (NNRTI)-resistance mutations such as K103N, V106M, Y188L and G190A, and importantly, the generation of a major clonally-amplified variant carrying K103N mutation in the mammary gland compartment (Figure 3). Thus, our data are in line with the likelihood that NVP-R variants preexisted prior to sdNVP prophylaxis in subject 4403. Together, these data identified i) the maternal breast milk as the source of the postnatally-transmitted NVP-R virus, and ii) NVP selection pressure as the underlying mechanism for the clonal amplification of a NVP-R variant in the breast milk compartment and subsequent transmission. Finally, the complete replacement of wild-type virus with NNRTI-resistant mutants was evident by 12 weeks postpartum coupled with a significant reduction of viral load in plasma and breast milk (Table 1); K103N mutation was present in 5 out of 8 (63%) pol sequences, whereas NNRTI resistance mutations V106M, Y188C or G190A were each present in 3 other sequences (Figure 3). Moreover, maternal week 12 viral variants were genetically heterogeneous and phylogenetically distinct from week 4 clonally-amplified variants; new clusters of near-identical *env* sequences (shown inside circles in Figure 4), indicated ongoing evolution and the transient nature of clonally-amplified variants as previously observed [17].

### Discussion and conclusions

Few studies have addressed the origin of NVP-R variants observed in postnatally-infected infants. Studies proposing transmission of NVP-R variants in infants based their conclusion on the results of a first time HIV positive test done at late times during the breastfeeding period under the assumption that NVP had been cleared from tissues [13,14]. Yet, the time interval between the infant HIV testing is usually broad in these studies, and the existing analyses did not include a time estimate since a MRCA of the variants using sequence diversity. Studies that have proposed transmission of wild-type virus followed by selection of resistant variants that were only detectable by sensitive assays based their interpretation on the timing of infection and likely exposure to NVP [10,11,23]. A similar interpretation should follow when population sequencing shows mixed wild-type and resistant viruses, unless both forms were simultaneously transmitted; a scenario less likely given the genetic bottleneck of MTCT of HIV [24,25] and the fact that most (~80%) HIV infections in heterosexuals [18,26] and infants via breast milk [27] result from transmission of a single virus. Thus, our study is important in that it establishes the NVP-R breast milk virus population selected by maternal sdNVP as the origin of the NVP-R virus in this postnatally-infected infant. However, our experimental design has a number of limitations. For instance, we cannot exclude the possibility that infection resulted from transmission of multiple identical viral genomes such as the progeny from a single infected cell in maternal milk, yet this limitation does not change our conclusion that the infant was infected by a NVP-R virus strain. Also, although we cannot exclude that the infecting virus strain in the infant could have been transmitted perinatally, our strict definition for breast milk transmission, i.e., HIV DNA PCR negative at birth and 4–6 weeks, but PCR positive at 12 weeks or later falls within the most likelihood for most cases of postnatal HIV transmission. However, these limitations do not impact the focus of this study which was to clarify whether NVP-R resistance is transmitted or arise de novo in the newly infected child. Antiretroviral treatment of an infant who harbors variants with resistance to a component of the antiretroviral treatment is more likely to fail therapy [28].

Breast milk HIV quasispecies include multiple independent lineages with evidence of intermixing with plasma virus [17,29-31], while some variants undergo clonal bursts of replication [17,30,31]. Clusters of monotypic and low-diversity variants have also been reported in blood and different body compartments such as the female genital tract [32], lung [33], semen [34], and cerebrospinal fluid [35]. Compartmentalization and local replication have been proposed as an explanation. Here, we propose that upon selection by NVP, blood NVP-R variants traffic into the mammary gland and seed the breast milk compartment. Next, a single NVP-R variant undergoes bursts of local replication, contributing a disproportionately large number of NVP-R virions in the milk virus pool and facilitating transmission of a drug-resistant virus via breastfeeding. The abundance of K103N clonal variants in the mother, and high-level viremia of the subtype C T/F K103N virus in the infant suggest that this mutation has little impact on viral fitness. Indeed, K103N mutation is one of the most common mutations selected by NVP as reported in previous MTCT studies [11,36]. Moreover, *in vitro* studies indicated that K103N mutation has no effect on replication fitness, while the Y181C mutation results in a much less fit virus when introduced in the backbone of the subtype C molecular clone MJ4 [37]. Our data demonstrate that a single antiretroviral drug, such as NVP provides a mechanism underlying the clonal amplification of resistant variant(s) in breast milk, thus increasing the transmission risk of drug-resistant viruses in infants. The role of ARV drugs in shifting the viral landscape toward the clonal amplification of drug-resistant variants deserves to be further investigated.

In summary, although we are reporting a single MTCT pair analysis, our data are in line with other studies that emphasize the need to implement MTCT prophylaxis strategies that reduce the risk of development and clonal amplification of drug resistant variants in maternal blood and breast milk to prevent MTCT of drug-resistant HIV variants. Our work suggests that administration of sdNVP prophylaxis can lead to postnatal transmission of selected NVP-R variants, and support the global implementation of effective multidrug maternal/infant prophylaxis strategies to prevent infant acquisition of drug-resistant HIV variants that will complicate pediatric ARV treatment strategies.

## Competing interest

The authors declare that they have no competing interest.

## Authors’ contributions

SP designed the investigations, led the overarching clinical study, and contributed to manuscript writing; MS performed maternal virus amplification and sequencing and sequence analysis; FG led infant virus sequencing and analysis; FC performed infant virus amplification and sequencing; GL performed the phylogenetic analysis; LK led patient enrollment and clinical site oversight; BH and GM contributed to sequence analysis and interpretation; JS-G led the virus sequencing, analysis, and interpretation and wrote the manuscript. All authors read and approved the final manuscript.

## Supplementary Material

Additional file 1 Material and methods. Click here for file
