# Supplementary material for: Clonal amplification and maternal-infant transmission of nevirapine-resistant HIV-1 variants in breast milk following single-dose nevirapine prophylaxis
Source: Retrovirology. 2013 Aug 14;10:88. doi: 10.1186/1742-4690-10-88 (PMC3765243; doi:10.1186/1742-4690-10-88)
Supplement: Additional file 1 — Material and methods. [file 1742-4690-10-88-S1.doc]

**Material and methods**

**Ethics statement**

Subject 4403 and her infant (4419) were participants of a study approved by the Division of Acquired Immunodeficiency Syndrome, National Institute of Allergy and Infectious Diseases, National Institute of Health (DAIDS-ES ID 10491); the College of Medicine Research and Ethics Committee in Malawi (P.06/06/440); and the institutional review board of Duke University (Pro00003582). Participant 4403 provided informed consent to be enrolled as a participant of the CHAVI 009 cohort of pregnant women testing HIV positive by a rapid antibody test from rural health clinics outside Blantyre, Malawi. Participant 4403 was offered perinatal single dose nevirapine (sdNVP) prophylaxis to prevent intrapartum HIV transmission to her child; she was also offered NVP administration to her child shortly after birth to prevent postnatal transmission. Written consent to include the infant in the study was obtained from the mother, and the father was informed.

**Sample collection and processing**

Subject 4403 was enrolled in the study at delivery, and gave birth to an uninfected child who was breastfed until his death at 6 months of age. Maternal blood was collected 4 weeks prior to delivery, at delivery, and 4 and 12 weeks postpartum. In addition, breast milk samples were obtained 4 and 12 weeks postpartum from left and right breasts that were free of cracked or bleeding nipples to rule out potential contamination of blood in breast milk samples. Plasma was isolated by centrifugation, and breast milk supernatant was isolated by centrifugation to separate the lipid and cellular portions. Child plasma was collected at birth, 4, and 12 weeks of age.

**Plasma and breast milk HIV load**

Virus loads were measured using the Roche Cobas Ampliprep/Cobas TaqMan 48 for HIV-1 viral load assay with limits of detection of 48 (plasma) and 240 (breast milk) HIV RNA copies/ml. Breast milk supernatant was diluted 1:5 in phosphate-buffered saline prior to analysis. The laboratory performing these assays was enrolled in the National Institute of Allergy and Infectious Diseases Division of AIDS Virology Quality Assessment Program and certified for HIV load determinations.

**Single genome amplification (SGA) and sequencing of HIV**

Plasma aliquots containing approximately 10,000 RNA copies or less were extracted using a QIAamp Viral RNA Mini kit (Qiagen). For plasma and breast milk supernatant samples with low viral loads, 1-ml aliquots were first centrifuged at 23,600 x g for 1 h at 4°C to concentrate the virus in approximately 100 µl of leftover volume. Pelleted virus was immediately used for RNA extraction. Depending on the virus load in plasma or milk, Between <240 and 10,000 RNA copies were reverse transcribed. HIV genes envelope (*env*) and polymerase (*pol*), half genomes, and 9 Kb fragments were PCR amplified by SGA assays as previously described using plasma and breast milk samples. HIV *pol* sequences were amplified with outer primers in a 1st round PCR forward 4SF1 (5’-AACTGTGGCAAGGAAGGGCACATAG-3’; nt 1969-1993) and reverse 4SR2 (5’-CTTGCCACACAATCATCACCTGCCAT-3’; nt 5052-5077), and inner primers in a 2nd round PCR forward POF3 (5’-GAAGGGCACATAGCCAGAAATTGCAG-3’; nt 1981-2006) and reverse POR4 (5’-CAATCATCACCTGCCATCTGTTTTCCATA-3’; nt 5040-5068). For amplification of infant’s 5’half genome, the primers for the 1st round PCR were 2010ForRC (5'-GTCTCTCTAGGTRGACCAGAT-3'; nt 456-476) and 2010Rev1C (5'-CTTCTTCCTGCCATAGGAAAT-3'; nt 5963-5980) and the primers for the 2nd round PCR were 2010ForR1C (5'-TAGGTRGACCAGATYWGAGCC-3'; nt 463-483) and 2010Rev2C (5'-AAGCAGTTTTAGGYTGRCTTCCTGGATG-3'; nt 5867-5894). For amplification of 3’-half genome, the primers for the 1st round PCR were 07For7 (5'-CAAATTAYAAAAAATTCAAAATTTTCGGGTTTATTACAG-3'; nt 4875-4912) and 2.R3.B6R (5'-TGAAGCACTCAAGGCAAGCTTTATTGAGGC-3'; nt 9607-9636), and the primers for the 2nd round PCR were VIF1 (5'-GGGTTTATTACAGGGACAGCAGAG-3'; nt 4900-4923) and Low2C (5'-TGAGGCTTAAGCAGTGGGTTCC-3'; nt 524-506). Amplicons were Sanger sequenced using BigDye Terminator v.3.1 chemistry in an ABI 3730xl DNA analyzer (Life Technologies). Sequences were edited using Sequencher program, version 5.1 (Gene Codes). Both strands of DNA were sequenced. All chromatograms were carefully inspected for sites of ambiguous sequence (double peaks).

**Phylogenetic sequence analysis**

Sequences were screened for significant evidence of APOBEC-driven G-to-A hypermutation, using the Sequence Database tool Hypermut 2.0 (http://www.hiv.lanl.gov/content/sequence/HYPERMUT/hypermut.html) from Los Alamos National Laboratory. HIV hypermutated sequences were excluded from subsequent analyses. Sequence alignments were produced using ClustalW and were subsequently adjusted to optimize codon alignment. Regions that could not be aligned unambiguously were omitted in further phylogenetic analyses but were included in all other genetic and phenotypic analyses of the virus populations. Neighbor-joining phylogenetic trees including *env* and *pol* sequences from each of the time points examined were constructed to visualize changes in the viral landscape such as clonal amplifications . Maximum-likelihood phylogenetic trees were inferred using PhyML, version 3.0 and phylogenetic support was based on 100 bootstrap replicates . Clonally-amplified variants in a chronically HIV-infected individual were defined as a group of low-diversity sequences that form clusters or rakes of identical and near-identical sequences that contrast with a more genetically heterogeneous viral population in a phylogenetic tree; these clonally-amplified variants coalesce to a consensus sequence and are either identical to or differed from the consensus by up to four nucleotide substitutions (prior to any gap stripping) indicating recent common ancestry. Recombination breakpoints in the sequence alignment for each subject were sought using GARD . We also used a mathematical model of random evolution to estimate the minimum number of days that would be required to explain the observed within-patient HIV-1 sequence diversification from a single most recent common ancestor using the Poisson-Fitter tool from the Los Alamos National Laboratory (http://www.hiv.lanl.gov/content/sequence/POISSON_FITTER/poisson_fitter.html), as previously described for *env* diversification .

Under the setting of recent infection and homogeneous sequences, which is the case for this mother-to-child transmission pair, this model assumes that the initial infecting strain replicates exponentially with no selection pressure. Under these simple circumstances, all sequences coalesce to the same founder, i.e., the most recent common ancestor, and the estimated time elapsed since this founder initiated infection can be accurately estimated by the Poisson model; we previously demonstrated that time estimates to a most recent common ancestor using the Poisson model were virtually identical to those estimated using the coalescent or Bayesian method, but the analyses are simpler and faster . BEAST analyses were conducted using software from version 1.7.5 of the BEAST package . An exponential relaxed clock model was used to estimate evolutionary rates using tip-dated maternal and infant derived sequences [mean = 2.06 x 10^-2^ substitutions/site/year (95% highest probability density interval 7.86 x 10^-3^ – 3.37 x 10^-2^)] and the days from most-recent common ancestor . To examine if HIV sequences conformed to model predictions, we first obtained the frequency distribution of all intersequence Hamming distances (defined as the number of base positions at which two sequences differ) and determined the chi-squared goodness of fit to test whether the Hamming distance distribution significantly diverges from a Poisson (small P-values indicate a bad fit).

**Drug resistance mutational analysis**

HIV *pol* sequences were screened for mutations conferring antiretroviral resistance using the Stanford University HIV Drug Resistance database (http://hivdb.stanford.edu/hiv). The search engine for HIVdb Program: Sequence Analysis can be found at http://sierra2.stanford.edu/sierra/servlet/JSierra?action=sequenceInput.

**Nucleotide sequence accession numbers**

HIV sequences determined in this study were deposited in GenBank under accession numbers HM070674-HM070790, HQ596118-HQ596136 and KF527060-KF527193.

**References**

1. Salazar-Gonzalez JF, Salazar MG, Keele BF, Learn GH, Giorgi EE, Li H, Decker JM, Wang S, Baalwa J, Kraus MH, et al: **Genetic identity, biological phenotype, and evolutionary pathways of transmitted/founder viruses in acute and early HIV-1 infection.** *J Exp Med* 2009, **206:**1273-1289.

2. Salazar-Gonzalez JF, Salazar MG, Learn GH, Fouda GG, Kang HH, Mahlokozera T, Wilks AB, Lovingood RV, Stacey A, Kalilani L, et al: **Origin and evolution of HIV-1 in breast milk determined by single-genome amplification and sequencing.** *J Virol* 2011, **85:**2751-2763.

3. Rose PP, Korber BT: **Detecting hypermutations in viral sequences with an emphasis on G --> A hypermutation.** *Bioinformatics* 2000, **16:**400-401.

4. Larkin MA, Blackshields G, Brown NP, Chenna R, McGettigan PA, McWilliam H, Valentin F, Wallace IM, Wilm A, Lopez R, et al: **Clustal W and Clustal X version 2.0.** *Bioinformatics* 2007, **23:**2947-2948.

5. Guindon S, Delsuc F, Dufayard JF, Gascuel O: **Estimating maximum likelihood phylogenies with PhyML.** *Methods Mol Biol* 2009, **537:**113-137.

6. Felsenstein J: **Confidence limits on phylogenies: an approach using bootstrap.** *Evolution* 1985, **39:**783-791.

7. Kosakovsky Pond SL, Posada D, Gravenor MB, Woelk CH, Frost SD: **Automated phylogenetic detection of recombination using a genetic algorithm.** *Mol Biol Evol* 2006, **23:**1891-1901.

8. Lee HY, Giorgi EE, Keele BF, Gaschen B, Athreya GS, Salazar-Gonzalez JF, Pham KT, Goepfert PA, Kilby JM, Saag MS, et al: **Modeling sequence evolution in acute HIV-1 infection.** *J Theor Biol* 2009, **261:**341-360.

9. Keele BF, Giorgi EE, Salazar-Gonzalez JF, Decker JM, Pham KT, Salazar MG, Sun C, Grayson T, Wang S, Li H, et al: **Identification and characterization of transmitted and early founder virus envelopes in primary HIV-1 infection.** *Proc Natl Acad Sci U S A* 2008, **105:**7552-7557.

10. Drummond AJ, Suchard MA, Xie D, Rambaut A: **Bayesian phylogenetics with BEAUti and the BEAST 1.7.** *Mol Biol Evol* 2012, **29:**1969-1973.

11. Drummond AJ, Ho SY, Phillips MJ, Rambaut A: **Relaxed phylogenetics and dating with confidence.** *PLoS Biol* 2006, **4:**e88.
